# Pre-slaughter, slaughter and post-slaughter defects of skins and hides at the Sheba Tannery and Leather Industry, Tigray region, northern Ethiopia

**DOI:** 10.4102/ojvr.v82i1.931

**Published:** 2015-08-21

**Authors:** Tesfay Kahsay, Guesh Negash, Yohannes Hagos, Birhanu Hadush

**Affiliations:** 1 College of Veterinary Medicine, Mekelle University, Ethiopia

## Abstract

Skins and hides are perishable resources that can be damaged by parasitic diseases and human error, which result in downgrading or rejection. This study was conducted to identify defect types and to determine their prevalence in pickled sheep and wet blue goat skins and wet blue hides. Each selected skin or hide was examined for defects in natural light and the defects were graded according to established quality criteria in Ethiopian standard manuals. Major defects were captured by digital photography. The major pre-slaughter defects included scratches (64.2%), cockle (*ekek*) (32.8%), wounds or scars (12.6%), lesions from pox or lumpy skin disease (6.1%), poor substance (5%), branding marks (2.3%) and tick bites (1.5%). The presence of grain scratches in wet blue hides (76.3%) was significantly higher than in pickled sheep (67.2%) and wet blue goat (59.1%) skins. The major slaughter defects included flay cuts or scores, holes, poor pattern and vein marks, with a higher occurrence in wet blue goat skins (28.7%; *P* < 0.001) than in wet blue hides (22.8%) and pickled sheep skins (11.1%). The most prevalent post-slaughter defects were grain cracks (14.9%), hide beetle damage (8%), damage caused by heat or putrefaction (3.7%) and machine-induced defects (0.5%). Grain cracks (27.04%) and hide beetle damage (13.9%) in wet blue goat skins were significantly more common than in wet blue hides and pickled sheep skins. These defects cause depreciation in the value of the hides and skins. Statistically significant (*P* < 0.001) higher rejection rates were recorded for wet blue hides (82.9%) than for pickled sheep skins (18.3%) and wet blue goat skins (8.5%). Improved animal health service delivery, effective disease control strategies and strong collaboration between stakeholders are suggested to enhance the quality of skins and hides.

## Introduction

Ethiopia has 53.4 million cattle, 25.5 million sheep and 22.7 million goats. These numbers illustrate a considerable potential for the leather industry in the country (Central Statistical Authority [CSA] 2011/2012). With an expected off-take rate of 33%, 35% and 7% for sheep, goats and cattle, respectively, Ethiopia is capable of supplying 16–18 million hides and skins per annum (Abadi 2000; Ethiopian Sheep and Goat Productivity Improvement Program [ESGPIP] 2009; Ministry of Agriculture and Rural Development 2008). The sheep skins have a fine grain and compact structure and goat skins are well known for their quality and international acceptance for producing various leather products.

Skins and hides are the most valuable export item for the country other than coffee (Engineering Capacity Building Program 2009), with export earnings for the country ranging from $405 to $590 million between 1998 and 2004 (Ayele *et al.* 2003; Tadesse 2005). The leather industry is one of the fastest-growing economic sectors in Ethiopia (Abadi 2000; Bayou 2007). The 26 operational tanneries in the country have a soaking capacity for 153 650 sheep and goat skins and 9725 cattle hides per day (United Nations Industrial Development Organization [UNIDO] 2008). However, they are not working to full capacity, as the hides and skins become available only when meat is needed and are not supplied for sustained leather processing (Bisrat 2013).

The leather industry processes raw hides and skins and produces semi-processed and finished leather both for export and for local markets (Abadi 2000). The semi-processed products are pickled sheep skin, wet blue goat skin and wet blue hides. Pickling refers to treating unhaired, limed, delimed and bated hides or skins with a solution of salt and acid (e.g. sulphuric acid or formic acid) to preserve them or prepare them for the tanning process. Wet blue skins or hides refer to products that have been chrome tanned but not dried (Quality Standard Authority of Ethiopia 2008).

However, the sector faces several challenges. Large numbers of hides and skins are discarded or their quality is substantially reduced by factors that can be avoided. Some of these factors are inherent to the production structure and animal husbandry practices, whereas others arise from the dispersal of the slaughter facilities, unfavourable marketing structures, poor handling (e.g. presentation and transportation) of the raw stock, and insufficient collection and preparation for further processing and export.

Defects are generally classified as pre-slaughter, slaughter, and post-slaughter defects (Leach 2002). The pre-slaughter defects include cockle (*ekek*), which is due to an allergic skin hypersensitivity reaction to parasitic infestation, grain scratches, pox lesions, warts, tick damage, branding, age (shrinkage) and poor substance (thickness of the skin or hide, toughness of the fibres and the closeness of the texture of the fibres). The major slaughter defects are flay cuts (scores), holes (a complete perforation of the skin or hide resulting from a knife or flaying appliance), poor pattern (an asymmetric skin or hide due to bad opening cuts or distortion during drying because of uneven tension), and vein marks (traces of blood vessels in the skin where the blood was not completely drained). Post-slaughter defects include heating or putrefaction (bacterial and enzymatic breakdown due to improper curing), hide beetle damage, machine damage and grain crack (ESGPIP 2009; Quality Standard Authority of Ethiopia 2008).

Another aspect in the recognition of the defects is that the quality of the hides and skins is seen only after additional costs have been incurred for the removal of hair or wool or when the tanning process has been completed. Raw hides and skins of high grades subsequently have to be rejected or downgraded (Kidanu 2001). In realising the development potential and economic importance of hides and skins, the government of Ethiopia has launched various development programmes aimed at increasing the supply and improving the quality of the raw material. The sector is, however, still constrained by poor quality of raw materials, lack of an efficient marketing structure, weak extension services, competition from local or rural tanning practices and a lack of price incentive for the production of good-quality raw materials (Yacob 2013).

According to the CSA (2011/2012), the Tigray region has 3.5 million head of cattle, 2.9 million goats and 1.1 million sheep. Despite the availability of substantial resources and active attempts to improve animal husbandry and health management, the leather sector contributes only marginally to the national economy so far. This is mainly due to the presence of livestock disease and difficulties to meet the strict international health and trade requirements set by the World Organisation for Animal Health (OIE) (CSA 2011/2012). Detailed information on the major causes of defects in hides and skins is urgently needed to design and implement a cost-effective and sound animal disease control strategy. The objectives of this study were to identify the major defect types in skins and hides and to determine their prevalence in pickled hides and skins of sheep and wet blue goat skins and hides at the Sheba Tannery and Leather Industry in Tigray, northern Ethiopia.

## Materials and methods

### Study area

The study was conducted at the Sheba Tannery and Leather Industry (13°47′N, 39°36′E), which is located in Wukro in eastern Tigray, a northern regional state of Ethiopia. It is about 830 km from the country's capital city, Addis Ababa, and 47 km from Mekelle, the capital city of the region. The tannery obtains raw sheep and goat skins and hides mainly from the Tigray region, as well as from Addis Ababa, Gondar, Wollo and the Amhara region (Ministry of Agriculture and Rural Development 2008). The tannery has a processing capacity of 6000 pieces of skin and 600 hides per day. The semi-processed sheep and goat skins and cattle hides include pickled skins, wet blue goat skins and wet blue hides. The tannery also produces 800 pairs of shoes daily (UNIDO 2008).

### Study design and data management

A cross-sectional study was conducted on skins and hides collected from different districts or zones of the Tigray region. Systematic stratified sampling was used, whereby only 20% of each delivered batch was randomly selected and considered in this study. Each selected skin or hide was examined for defects in natural light by trained skin selectors of the company and the research groups. The defects were identified and graded according to the quality standards as indicated by the Quality Standard Authority of Ethiopia (2008). Defects were classified according to three major groups: pre-slaughter, slaughter and post-slaughter defects. A spreadsheet was used to create a database and STATA software (version 11.0) was used to analyse the data. Descriptive statistics were used to summarise the data with regard to frequencies and percentage. Bivariate logistic regression analysis was performed to quantify the degree of association between the risk factors and defects, which was expressed as an odds ratio (OR) and a 95% confidence interval (CI). A significance level of 0.05 was used to determine statistically significant differences in all analyses.

## Results

The defects resulting in hides or skins being downgraded or rejected at the study site were classified as pre-slaughter, slaughter and post-slaughter defects.

The major pre-slaughter defects in pickled sheep skins (Table 1) were cockle (68.0%), scratches (67.2%), scars (11.05%) and poor substance (9.8%). The major slaughter defects were flay cuts or scores (11.1%), holes (6.7%) and poor pattern (0.5%). The most prominent post-slaughter defect in pickled sheep skins was heat damage or putrefaction (3.7%).

**TABLE 1:** Pre-slaughter, slaughter and post-slaughter defects observed in pickled sheep skins from the Tigray region, Ethiopia.

Defect subtype	Defect type					
		Pre-slaughter (N = 4534)		Slaughter (N = 3045)		Post-slaughter (N = 3045)	
		n	%	n	%	n	%
Scratch	3045	67.2	-	-	-	-
Pox	54	1.2	-	-	-	-
Scar/Wound	501	11.0	-	-	-	-
Cockle/ekek	3085	68.0	-	-	-	-
Branding mark	3	0.1	-	-	-	-
Age	62	1.4	-	-	-	-
Poor substance	444	9.8	-	-	-	-
Warts	2	0.04	-	-	-	-
Flay cuts/scores	-	-	504	11.1	-	-
Holes	-	-	204	6.7	-	-
Poor pattern	-	-	14	0.5	-	-
Heating/Putrefaction	-	-	-	-	106	3.5
Machine defects	-	-	-	-	0	0
Hide beetle damage	-	-	-	-	0	0
Grain crack	-	-	-	-	0	0

The major pre-slaughter defects of wet blue salted and dry goat skins (Table 2) were scratches (59.1%) and pox lesions (10.8%), whereas slaughter defects included flay cuts and scores (28.7%), holes (5.6%) and poor pattern (3.7%).Post-slaughter defects observed in both wet blue salted and dry goat skins were putrefaction and machine damage. Grain cracks (49.1%) and hide beetle damage (25.2%) were observed only in dry goat skins.

The major pre-slaughter defects seen in wet blue hides (Table 3) were caused by grain scratches (79.4%), scars (66.1%), branding (28.8%), tick-bite damage (22.7%), age or shrinkage (12.5%), and lumpy skin disease (LSD) (8.3%). The slaughter defects of hides included flay cuts or scores (22.8%), holes (17.5%) and vein marks (11.5%). Putrefaction (21.8%) and hide beetle damage (4.6%) were the only post-slaughter defects seen on hides.

**TABLE 2:** Pre-slaughter, slaughter and post-slaughter defects of wet blue salted and dry goat skins from the Tigray region, Ethiopia.

Defect subtype	Defect type																	
		Pre-slaughter						Slaughter						Post-slaughter					
		Salted skins (N = 2014)		Dry skins (N = 2468)		Total (N = 4482)		Salted skins (N = 2014)		Dry skins (N = 2468)		Total (N = 4482)		Salted skins (N = 2014)		Dry skins (N = 2468)		Total (N = 4482)	
		n	%	n	%	n	%	n	%	n	%	n	%	n	%	n	%	n	%
Scratch	1428	71.2	1220	49.4	2648	59.1	-	-	-	-	-	-	-	-	-	-	-	-
Pox	313	15.5	169	6.8	482	10.8	-	-	-	-	-	-	-	-	-	-	-	-
Scar/Wound	185	9.2	91	3.7	276	6.1	-	-	-	-	-	-	-	-	-	-	-	-
Cockle/ekek	36	1.8	7	0.3	43	1.0	-	-	-	-	-	-	-	-	-	-	-	-
Branding mark	20	1.0	16	0.6	36	0.8	-	-	-	-	-	-	-	-	-	-	-	-
Age	6	0.3	0	0	6	0.1	-	-	-	-	-	-	-	-	-	-	-	-
Poor substance	15	0.7	20	0.8	35	0.8	-	-	-	-	-	-	-	-	-	-	-	-
Warts	5	0.2	9	0.4	14	0.3	-	-	-	-	-	-	-	-	-	-	-	-
Flay cuts/scores	-	-	-	-	-	-	481	24.0	806	32.7	1287	28.7	-	-	-	-	-	-
Holes	-	-	-	-	-	-	90	4.5	162	6.6	252	5.6	-	-	-	-	-	-
Poor pattern	-	-	-	-	-	-	68	3.4	99	4	167	3.7	-	-	-	-	-	-
Heating/Putrefaction	-	-	-	-	-	-	-	-	-	-	-	-	34	1.7	19	0.8	53	1.2
Machine defects	-	-	-	-	-	-	-	-	-	-	-	-	28	1.4	11	0.4	39	0.9
Hide beetle damage	-	-	-	-	-	-	-	-	-	-	-	-	0	0	623	25.24	623	13.9
Grain crack	-	-	-	-	-	-	-	-	-	-	-	-	0	0	1212	49.1	1212	27.1

**TABLE 3:** Pre-slaughter, slaughter and post-slaughter defects of wet blue hides from cattle in the Tigray region, Ethiopia.

Defect subtype	Defect type					
		Pre-slaughter (N = 633)		Slaughter (N = 633)		Post-slaughter (N = 633)	
		n	%	n	%	n	%
Scratch	506	79.4	-	-	-	-
LSD	55	8.3	-	-	-	-
Scar/Wound	438	66.1	-	-	-	-
Cockle	37	5.6	-	-	-	-
Branding mark	791	28.8	-	-	-	-
Age	79	12.5	-	-	-	-
Warts	1	0.2	-	-	-	-
Ticks	144	22.7				
Flay cuts/scores	-	-	151	22.8	-	-
Holes	-	-	111	17.5	-	-
Vein marks			73	11.5		
Heating/Putrefaction	-	-	-	-	138	21.8
Hide beetle damage	-	-	-	-	29	4.6
Machine defects	-	-	-	-	0	0
Heat	-	-	-	-	0	0
Cracks	-	-	-	-	0	0

LSD, lumpy skin disease.

### Pre-slaughter defects

**FIGURE 1:**
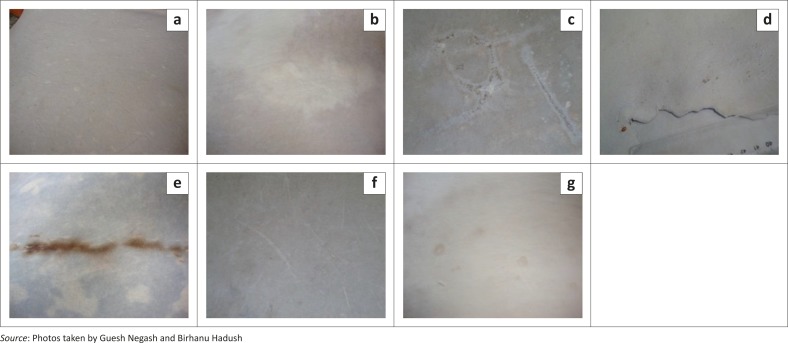
Major pre-slaughter defects seen in hides and skins: (a) pox lesions; (b) cockle (*ekek*); (c) branding marks; (d) tick damage; (e) aged skins; (f) grain scratch; (g) wart lesions.

A total of 9649 hides and skins were examined for pre-slaughter defects, of which 46.5% were from wet blue goat skins, 47.0% from pickled sheep skins and 6.5% from wet blue hides. Examples of pre-slaughter defects are shown in Figure 1.

The presence of grain scratch in wet blue hides (76.3%) was significantly higher than in pickled sheep (67.2%) and wet blue goat skins (59.1%). Compared with wet blue goat skins, wet blue hides and pickled sheep skins were respectively found to be 2.8% and 1.4% more likely to be damaged by scratches (for wet blue hides: OR = 2.75, 95% CI: 2.251–3.382; for pickled skins: OR = 1.4, 95% CI: 1.299–1.543; *P* < 0.001). Cockle lesions were seen mainly on pickled sheep skins (68.0%) and wet blue hides (5.7%), with only 1.0% of wet blue goat skins being affected. Pickled sheep skins were more than 200 times more likely to be affected by cockle than wet blue goat skins and the difference was statistically significant (OR = 219.78, 95% CI: 161.726–298.693, *P* < 0.001). Comparable defects due to pox were observed more often on wet blue goat skins (10.8%) and wet blue hides (8.1%) than in pickled sheep skins (1.2%). Wound or scar damage was seen more often in cattle hides (66.1%) than in sheep (11.0%) and goat (6.1%) skins. This defect was statistically significant and wet blue hides were 34.22 times more likely to be affected than wet blue goat skins (OR = 34.22, 95% CI: 27.798–42.147, *P* < 0.001). Wet blue hides and pickled sheep skins were, respectively, 22% and 90% less likely to be affected by pox or LSD virus than wet blue goat skins (for wet blue hides: OR = 0.78, 95% CI: 0.589–1.057; for pickled skins: OR = 0.10, 95% CI: 0.075–0.132, *P* < 0.001). Defects due to branding were more common on wet blue hides (28.8%) than on wet blue goat skins (0.8%) and pickled sheep skins (0.1%). This difference was statistically significant, with wet blue hides almost 50 times more likely to have branding marks than wet blue goat skins (OR = 49.83, 95% CI: 34.411–72.18,* P* < 0.001). Defects caused by poor substance in pickled sheep skins (9.8%) were significantly more common (*P* < 0.001) than in wet blue goat skins (0.8%) and wet blue hides (0%). Tick damage was observed only in wet blue hides (22.7%). The presence of warts was limited in both skins and hides.

### Slaughter defects

**FIGURE 2:**
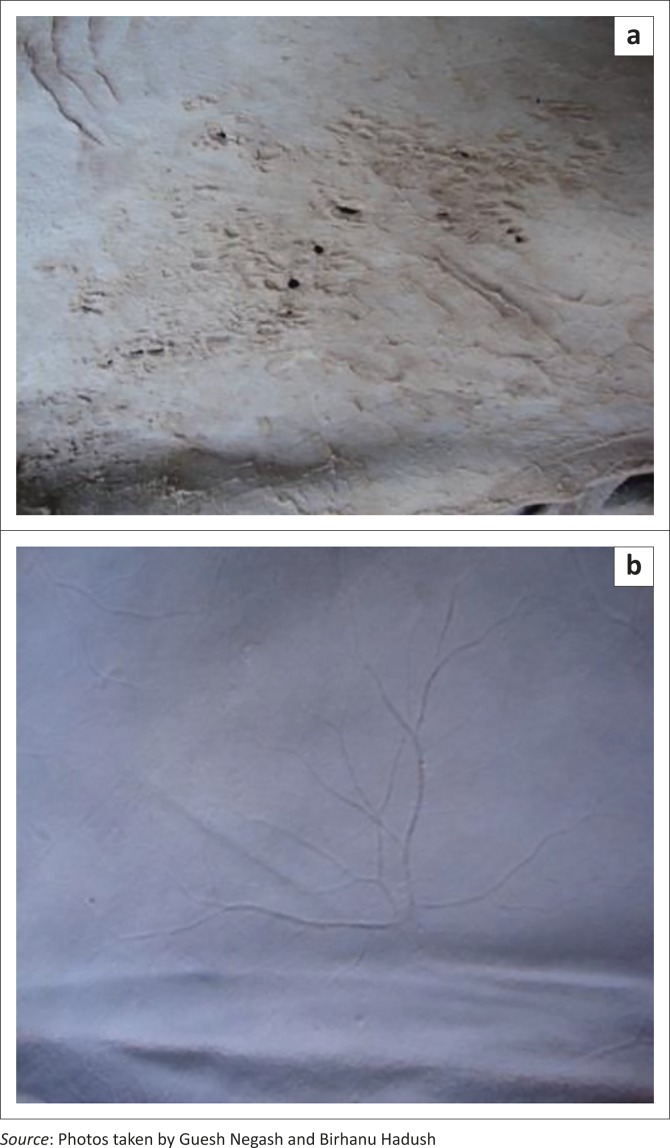
Slaughter defects seen in hides and skins: (a) flay cuts or scores and (b) vein marks.

A total of 8160 skins and hides were examined for slaughter defects, of which 54.9% were wet blue goat skins, 37.3% pickled sheep skins and 7.8% wet blue hides. Examples of slaughter defects are shown in Figure 2.

The major slaughter defects were flay cuts or scores (23.8%), holes (7.0%), and poor pattern and vein marks (0.9%). Defects due to flay cuts and scores occurred significantly more often (*P* < 0.001) in wet blue goat skins (28.7%) than in wet blue hides (22.8%) or pickled sheep skins (11.1%), which were respectively 23% and 51% less likely to be damaged by knife cuts than wet blue goat skins (for wet blue hides: OR = 0.77, 95% CI: 0.640–0.944; for pickled skins: OR = 0.49, 95% CI: 0.438–0.552). Holes were the major defect in wet blue hides (17.5%) and more common than in the skins of pickled sheep (6.7%) and goats (5.6%). A higher proportion (*P* < 0.001) of poor pattern was seen on wet blue goat skins (3.7%) than on pickled sheep skins (0.5%) or wet blue hides (0.0%). Wet blue hides presented with vein marks (11.5%), whereas no vein mark defects were observed on wet blue goat skins and pickled sheep skins.

### Post-slaughter defects

**FIGURE 3:**
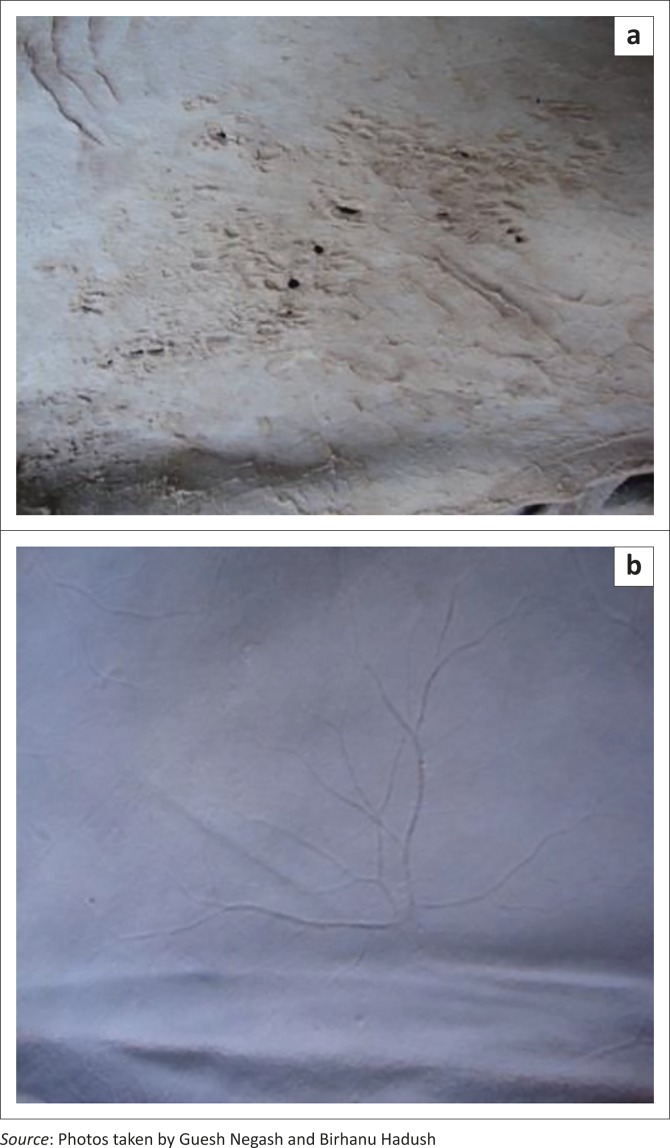
Post-slaughter defects seen in hides and skins: (a) holes and putrefaction; (b) grain crack and (c) beetle damage.

A total of 8160 skins and hides were examined for post-slaughter defects. Examples of post-slaughter defects are shown in Figure 3.

Of the total number of skins and hides examined for post-slaughter defects, 54.9% were wet blue goat skins, 37.3% were pickled sheep skins and 7.8% were wet blue hides. The defects observed in these products were caused by grain cracks (14.9%), hide beetle damage (8%), heat or putrefaction (3.7%) and machine damage (0.5%). Grain crack was the major post-slaughter defect in wet blue goat skins (27.04%). Severe damage was caused by hide beetles in wet blue goat skins (13.9%) and in wet blue hides (4.6%). No beetle damage was found in pickled sheep skins. Putrefaction of hides (21.8%) was significantly more common than in wet blue goat and sheep skins (1.1% and 0.1%, respectively). Post-slaughter defects observed in pickled sheep and wet blue goat skins included damage caused by heat or putrefaction (3.5%) and machine processing (0.9%), respectively.

### Quality of skins and hides

Damage due to single or combined defects was responsible for skins and hides being downgraded or rejected, with 82.9% of wet blue hides, 18.3% of pickled sheep skins and 8.5% of wet blue salted and dry goat skins being rejected (Table 4).

**TABLE 4:** Quality grade of semi-processed skins and hides at the Sheba Tannery and Leather Industry, Tigray, Ethiopia.

Product type	Size	Number of skins	Grade result				
						I-III	IV	V	VI	Reject
Pickled sheep skins	Small	1231	17	144	404	368	298
	Medium	9189	126	880	3537	3009	1637
	Large	7417	18	232	2520	3239	1408
	Extra large	4555	-	97	1817	1890	751
	Total	22 392	161 (0.78%)	1353 (6.04%)	8278 (36.97%)	8506 (37.99%)	4094 (18.30%)
Wet blue salted and dry goat skins	Small	6139	664	1093	3040	1093	249
	Medium	8861	1236	2165	3516	1489	455
	Large	3096	154	473	1552	917	465
	Extra large	3400	44	302	1225	1182	647
	Total	21 496	2098 (9.76%)	4033 (18.76%)	9333 (43.42%)	4681 (21.78%)	1816 (8.50%)
Wet blue hides	Small	196	-	-	8	44	144
	Medium	642	-	-	-	112	530
	Large	958	-	-	11	132	815
	Extra large	1436	-	6	41	199	1190
	Total	3232	-	6 (0.19%)	60 (1.86%)	487 (15.09%)	2679 (82.90%)

Grade I-III: hide or skin with good quality to minor defect; Grade IV: hide or skin with moderate defect; Grade V: hide or skin with severe defect; Grade VI: hide or skin with very severe defect; Rejection: hide or skin with 50% of its size unusable.

There was a statistically significant difference in rejection rate across the three product types. Wet blue hides and pickled sheep skins were, respectively, 52.49 and 2.42 times more likely to be rejected than wet blue goat skins (for wet blue hides: OR = 52.49, 95% CI: 47.342–58.218; for pickled skins: OR = 2.42, 95% CI: 2.286–2.571; *P* < 0.001). Grain scratch resulted in the downgrading and rejection of 67.2%, 71.2%, 49.4% and 72.3% of pickled sheep skin, wet blue or salted goat skin, wet blue or dry goat skin, and wet blue hides, respectively (data not shown). Pox accounted for 1.5%, 15.5%, 6.8% and 8.3% of pickled sheep skins, wet blue or salted goat skins, wet blue or dry goat skins and wet blue hides, respectively, being rejected. Cockle caused rejection in 68.0%, 1.8%, 0.3% and 5.6% of pickled sheep skin, wet blue or salted goat skin, wet blue or dry goat skin and wet blue hides, respectively. Branding caused rejection in 0.05%, 0.9%, 0.6% and 28.8% of pickled sheep skins, blue or salted goat skins, wet blue or dry goat skins and wet blue hides, respectively. Cracked grain resulted in rejection in 49.1% wet blue or dry goat skin. Hide beetle damage caused downgrading or rejection of 25.2% and 4.4% of wet blue or dry goat skins and wet blue hides, respectively.

## Discussion

The production of good-quality leather depends on the quality of raw stock. Defects in leather are costly from a production point of view and greatly reduce the sale value of the leather. Damage and defects can occur from birth to the completion of the leather processing (Leach & Wilson 2009). The common pre-slaughter defects seen in pickled sheep skins, wet blue goat skins and wet blue hides in this study included scratches, cockle (*ekek*), poor substance, brandings, and wounds and scars resulting from pox, LSD or tick infestations. These observations are in agreement with those of a study by Kidanu (2001). However, Tadesse and Mebrahitu (2010) reported a lower percentage of scratches, cockle and scars on pickled sheep skins and wet blue goat skins as the major pre-slaughter defects in the Sheba tannery. Similarly, Mulugeta (2008) reported lower rates of scratches, cockle and scars on pickled sheep skins and wet blue hides. In contrast, Ermias (2000) and Tefera and Abebe (2007) reported much higher presence of cockle on pickled sheep skins from the Sebeta and Dessie tanneries. Bisrat (2013) indicated that the occurrence of cockle was higher in sheep than in cattle and goats. This difference may be due to the variation in veterinary interventions in different districts, which may not be effective against the cockle-causing ectoparasites (especially lice and keds) in the study area.

Pox and LSD lesions were observed on wet blue goat skins, wet blue hides and pickled sheep skins. The animals infected by pox or the LSD virus develop blisters with small red spots, followed by the appearance of papules, vesicles, pustules and pox lesions, or deep, yellow, pea-sized oval ulcers that cause permanent scarring (Boden 2005). According to a report by ESGPIP (2009) and a study by Mulugeta (2008) pox and LSD lesions were also seen on wet blue goat skins, wet blue hides and pickled sheep skins. However, Ermias (2000) and Tefera and Abebe (2007) reported low rates of pox and LSD, which could be attributed to agro-ecological differences. Defects due to brand markings were more common on wet blue hides than on wet blue goat skins and pickled sheep skins. Tadesse (2005) also reported similar findings. Tick damage was observed only in wet blue hides, which is in agreement with the findings of Hagos, Yacob and Mulugeta (2013). The absence of tick lesions in skins could be due to effective ectoparasite control in small ruminants.

The major slaughter defects found in this study were flay cuts or scores, holes, poor pattern and vein markings. Flay cuts and scores are caused by the careless use of a knife during skinning of the carcass because of poor technique (ESGPIP 2009). This was more common on wet blue goat skins than on wet blue hides and pickled sheep skins. This finding is in agreement with previously reported findings (Berhanu *et al.* 2011; ESGPIP 2009; Yacob 2013). However, Bisrat (2013) found a higher number of flay cut defects in cattle compared with goats and sheep. Wet blue hides presented with holes more often than pickled sheep and goat skins. Similarly, a report by ESGPIP (2009) notes holes as major defects in wet blue hides. Vein marks were observed only in wet blue hides and are caused by incomplete bleeding of the carcass. Blood that remains in the blood vessels leads to the multiplication of bacteria (Yacob 2013).

The post-slaughter defects observed in this study included grain crack, hide beetle damage, and putrefaction, heat or machine defects. Similar post-slaughter defects in hides and skins have been reported previously (ESGPIP 2009). Grain crack was observed only in wet blue goat skins. Grain crack refers to broken fibres on the grain side of hides and skins that were dried before folding. Defects due to hide beetle damage were found in wet blue goat skins and wet blue hides, which is in agreement with the findings reported by Bisrat (2013). Putrefaction was more common in hides than in wet blue goat and sheep skins. This finding was not in line with the report of Yacob (2013). Most of the post-slaughter defects are due to improper curing or preservation and storage of hides and skins.

The mentioned defects lead to downgrading or rejection of skins and hides. In this study, a large proportion of wet blue hides were found to be rejected, followed by pickled sheep skins and wet blue goat skins (Table 4). At the Sheba tannery, cockle led to a financial loss of close to $800 000, with a further loss of close to $250 000 due to rejection of pickled sheep and wet blue goat skins in 2010 (Hagos *et al.* 2013). The estimated financial losses caused by defects stem mainly from the purchase of raw skins or hides that are of undetectable inferior quality, the cost of processing these skins or hides and the eventual downgrading of such skins or hides, thereby rendering them unsuitable for sale in export markets (Bayou 2005). Berhanu *et al.* (2011) documented that the major defects leading to skin rejection at the Modjo tannery were cockle and scratches, goat pox, poor substance, scars, and technical defects such as heat and knife cuts.

## Conclusion

It is not realistic to expect animal hides or skins to be perfect and defects are almost always present to some extent. Such defects cause depreciation in the value of the hides and skins and the consequence is that farmers, traders and the tanning industry suffer considerable financial losses. We found pre-slaughter defects to be more common than slaughter and post-slaughter defects combined. To improve the quality of hides and skins, animal health service delivery needs to be strengthened and there is a need for effective strategic planning, monitoring and assessment of disease control programmes. Awareness creation, training, and increased collaboration between the Bureau of Agriculture, international and regional organisations, research institutions and the Ethiopian leather associations can address unnecessary losses in the leather industry.
